# A Rare Case of Lateral Canthal Gouty Tophus Presenting as an Eyelid Mass

**DOI:** 10.1155/2016/9786810

**Published:** 2016-11-06

**Authors:** Austin S. Nakatsuka, Timothy F. McDevitt, Pamela S. Tauchi-Nishi

**Affiliations:** ^1^University of Hawaii John A. Burns School of Medicine, Honolulu, HI, USA; ^2^Department of Surgery, University of Hawaii John A. Burns School of Medicine, Honolulu, HI, USA; ^3^Department of Pathology, University of Hawaii John A. Burns School of Medicine, Honolulu, HI, USA; ^4^Queen's Medical Center, Honolulu, HI, USA

## Abstract

A 41-year-old man with a history of gout presented with an enlarging eyelid growth. Clinical examination revealed a mildly indurated nodule at the lateral canthus. Following resection, histopathological examination revealed needle-shaped, crystalline material surrounded by multinucleated giant cells, findings consistent with gouty tophus. This represents just the sixth case of gouty tophus of the eyelid reported in the literature.

## 1. Introduction

Gout is a complex disorder characterized by elevated levels of monosodium urate. Deposition of urate crystals in joint soft tissues can lead to a chronic inflammatory reaction producing debilitating deformities called tophi [1]. While tophi more commonly occur in the digits, ear pinna, prepatellar bursa, and olecranon, they very rarely occur around the eye [1–3]. Unusual cases of urate crystal deposition in the cornea, orbit, iris, and anterior chamber have been described in the literature [4–6]. We report an unsuspected case of gouty tophus involving the lateral canthus of the eyelid. To our knowledge, this is only the sixth case of gouty tophus of the eyelid reported in the literature. All patient information and images presented in this case report have been deidentified in accordance with the Health Insurance Portability and Accountability Act guidelines.

## 2. Case Report

A 41-year-old man with a medical history significant for congestive heart failure presented with a gradually enlarging growth of the right outer lid. The growth had developed continuously over 1 year and was now becoming mildly irritating and pruritic although it remained painless. Previous ophthalmic history was unremarkable.

The patient had a 10-year history of gout treated with daily allopurinol and colchicine as needed for periodic attacks. The patient revealed that he had a gouty mass on his ankle and another nodule on his toe. A subsequent uric acid level was elevated at 10.4 mg/dL. Family history was negative for gout.

On examination, visual acuity was 20/30-1 OU. Slit lamp examination revealed 2+ dermatochalasis with steatoblepharon and a white subcutaneous, mildly indurated nodule (4 × 7 × 4 mm) at the lateral canthus of the right eye. On clinical appearance, the mass was suspected to be a simple epidermal inclusion cyst. Examination of the conjunctiva, cornea, anterior chamber, and pupils demonstrated no abnormalities. Resection of the canthal tumor at the lateral palpebral raphe with subsequent periramal repair was done. The specimen was fixed in formalin and submitted for histopathological examination.

## 3. Histopathological Examination

**Figure 1:**
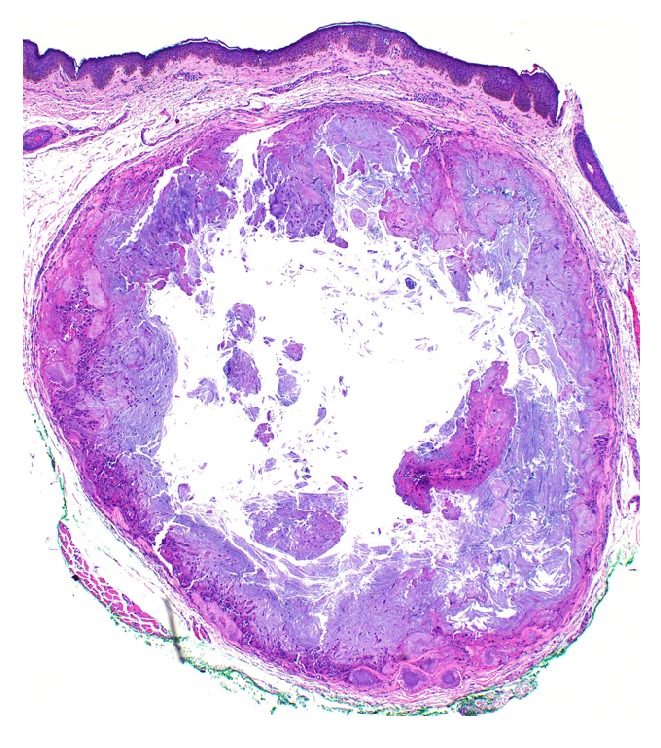
Light microscopic view of eyelid skin section demonstrating granulomatous reaction surrounding cavity of cystic structure containing crystalline material (hematoxylin-eosin stain, ×4).

**Figure 2:**
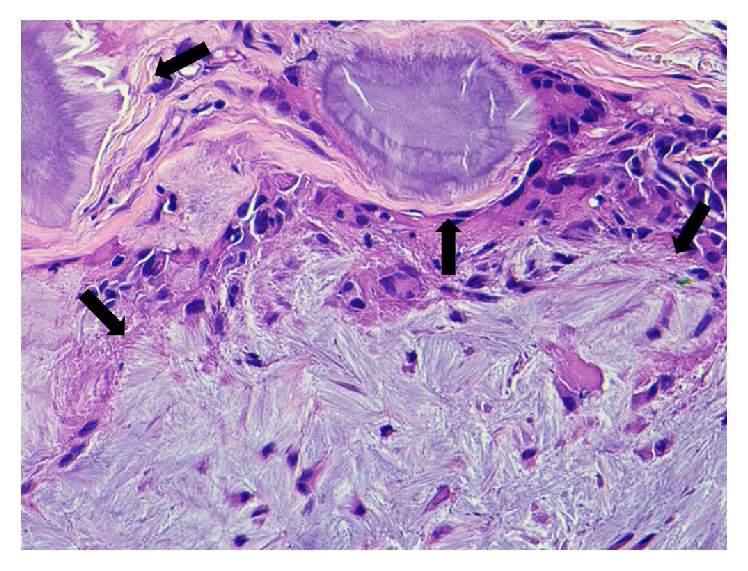
Histiocytes and multinucleated giant cells adjacent to urate crystals (arrows) with parallel, needle-like spokes in a radial pattern (hematoxylin-eosin stain, ×20).

Gross examination of the formalin-fixed specimen revealed skin with an underlying cystic structure (4 × 4 mm) containing soft white material. Histologic examination revealed eyelid skin with dermal deposits of amorphous eosinophilic material surrounded by a foreign body granulomatous reaction (Figure 1). Higher power magnification revealed needle-shaped, crystalline material surrounded by multinucleated giant cells and histiocytes (Figure 2).

## 4. Discussion

The appearance of ocular tophi is one of many ocular abnormalities that can occur in patients with gout. Rare cases have been reported of urate crystal deposition in the conjunctiva, sclera, corneal stroma and epithelium, anterior chamber, and iris [11]. A study by Ferry et al. reported that the most common ocular complication of gout is chronic bilateral ocular redness caused by conjunctival/episcleral hyperemia [4]. Lin et al. found that gout can lead to subconjunctival vesicles, subconjunctival hemorrhage, and scleral vascular tortuosity [11]. Very rare reports of uveitis due to gout have also been reported [4]. Elevated intraocular pressure has been claimed to be more prevalent in gout patients but little evidence has emerged to support it [4].

A literature search revealed only five prior cases of tophi in the eyelid or canthi (four at the medial/lateral canthi, one in the superior eyelid) reported between 1986 and 2008 (Table 1) [2, 7–10]. Documented in the French language, De Monteynard et al. described a case of a lateral canthus tophus in a 62-year-old patient that had the clinical appearance of a chalazion [7]. In the English literature, all cases of eyelid tophi were painless masses with no discharge, inflammation, or bleeding. These patients had previous histories of gout ranging from 3 to 20 years, with tophi presentation in areas such as the elbows and first metatarsal. Morris and Fleming encountered a right lateral canthal tophus developing over 1 year in a 44-year-old patient [8]. Yen et al. noted a right medial canthal tophus developing over 3 months in a 27-year-old patient [2]. Jordan et al. reported a case of left medial canthal tophus growing slowly over 2 years in a 68-year-old patient [9]. Yang et al. described a tophus in the middle superior eyelid enlarging over 9 years in a 64-year-old patient [10]. In the case described by Jordan et al. the tophus was skin-colored and resembled a basal cell carcinoma, based on the appearance of central depression and superficial crusting [9]. In contrast, the cases reported by Yang et al. and Morris and Fleming were yellow subcutaneous masses [8, 10]. Yen et al. described their tophus case as a chalky, white mass [2]. Our case presented as a white subcutaneous mass resembling an epidermal inclusion cyst. Despite the rarity in presentation, gouty tophi should be considered in the differential diagnosis of periocular masses in patients with a history of gouty lesions, especially if located within the canthi.

## Figures and Tables

**Table 1:** Case reports of eyelid and canthal tophi.

Reference	Patient age (years)	Duration of gout (years)	Duration of lesion (months)	Lesion location	Lesion size (MM)	Other lesions
De Monteynard et al. (1986) [7]	62	—	—	Lateral canthus	—	—
Morris and Fleming (2003) [8]	44	—	12	R lateral canthus	6 × 5 × 4	—
Yen et al. (2005) [2]	27	3	3	R medial canthus	11 × 5 × 5	L first metatarsal
Jordan et al. (2008) [9]	68	20	24	L medial canthus	5 × 6 × 4	L elbow, R elbow
Yang et al. (2008) [10]	64		108	L middle superior eyelid	13.9 × 9.4 × 7.6	—
Our patient	41	10	12	R lateral canthus	4 × 7 × 4	Ankle, toe
